# Synthesis and Antifungal Evaluation of 1-Aryl-2-dimethyl- aminomethyl-2-propen-1-one Hydrochlorides

**DOI:** 10.3390/molecules16064660

**Published:** 2011-06-03

**Authors:** Ebru Mete, Halise Inci Gul, Sinan Bilginer, Oztekin Algul, Mehmet Emin Topaloglu, Medine Gulluce, Cavit Kazaz

**Affiliations:** 1 Department of Chemistry, Faculty of Sciences, Ataturk University, 25240, Erzurum, Turkey; 2 Department of Pharmaceutical Chemistry, Faculty of Pharmacy, Ataturk University, 25240, Erzurum, Turkey; 3 Department of Pharmaceutical Chemistry, Faculty of Pharmacy, Mersin University, 33169, Mersin, Turkey; 4 Department of Biology, Faculty of Sciences, Ataturk University, 25240, Erzurum, Turkey

**Keywords:** acetophenone, antifungal activity, conventional heating, mannich bases, synthesis, microwave irradiation

## Abstract

The development of resistance to current antifungal therapeutics drives the search for new effective agents. The fact that several acetophenone-derived Mannich bases had shown remarkable antifungal activities in our previous studies led us to design and synthesize some acetophenone-derived Mannich bases, **1-8** and 2-acetylthiophene-derived Mannich base **9**, 1-aryl-2-dimethylaminomethyl-2-propen-1-one hydrochloride, to evaluate their antifungal activities. The designed chemical structures have *α*,*β*-unsaturated ketone moieties, which are responsible for the bioactivities of the Mannich bases. The aryl part was C_6_H_5_ (1); 4-CH_3_C_6_H_4_ (2); 4-CH_3_OC_6_H_4_ (3); 4-ClC_6_H_4_ (4); 4-FC_6_H_4_ (5); 4-BrC_6_H_4_ (6); 4-HOC_6_H_4_ (7); 4-NO_2_C_6_H_4_ (8); and C_4_H_3_S(2-yl) (9). In this study the designed compounds were synthesized by the conventional heating method and also by the microwave irradiation method to compare these methods in terms of reaction times and yields to find an optimum synthetic method, which can be applied for the synthesis of Mannich bases in further studies. Since there are limited number of studies reporting the synthesis of Mannich bases by microwave irradiation, this study may also contribute to the general literature on Mannich bases. Compound **7** was reported for the first time. Antifungal activities of all compounds and synthesis of the compounds by microwave irradiation were also reported for the first time by this study. Fungi (15 species) were used for antifungal activity test. Amphotericin B was tested as an antifungal reference compound. In conclusion, compounds **1-6**, and **9**, which had more potent (2–16 times) antifungal activity than the reference compound amphotericin B against some fungi, can be model compounds for further studies to develop new antifungal agents. In addition, microwave irradiation can be considered to reduce reaction period, while the conventional method can still be considered to obtain compounds with higher reaction yields in the synthesis of new Mannich bases.

## 1. Introduction

Mannich bases are generally formed by the reaction between formaldehyde, a secondary amine and a compound containing reactive hydrogen atoms. On occasion, aldehydes other than formaldehyde may be employed and the secondary amine may be replaced by ammonia and primary amines. This process is known as the Mannich reaction [1]. Mannich bases display varied biological activities such as antimicrobial [2,3,4,5], cytotoxic [6,7,8,9,10,11,12,13], anticancer [1,14,15], anti-inflammatory [16,17] and anticonvulsant [18,19] and DNA topoisomerase I inhibiting properties [20,21]. A Mannich base having at least one hydrogen atom at the *β*-position of amine group can undergo a deamination process to generate an *α,β*-unsaturated ketone moity. The biological activities of Mannich bases have mostly been attributed to *α,β*-unsaturated ketones [1,15]. A Mannich base itself may have this chemical moity or can generate it by the deamination process *in vivo* or under simulated conditions *in vitro* if the chemical structure of the compound permits this. *α,β*-Unsaturated ketones can alkylate nucleophiles, especially thiols, for bioactivity [7,15,22,23,24,25].

Primary and opportunistic fungal infections continue to increase rapidly because of the increased number of immune compromised patients such as AIDS, cancer and transplants [26]. The development of resistance to current antifungal therapeutics continues to drive the search for more effective new agents. It is reported that Mannich bases such as Mannich bases of conjugated styryl ketones [22], isatin N-Mannich bases [27], bis(*β*-aroylethyl)methyl or ethyl amine hydrochlorides and 3-aroyl-4-aryl-1-(methyl or ethyl or phenethyl)-4-piperidinol hydrochlorides have antifungal activity [2,4,5]. 

The fact that acetophenone-derived several Mannich bases had shown remarkable antifungal activities in our previous studies led us to design and synthesize some acetophenone-derived Mannich bases having acrylophenone structures, namely 1-aryl-2-dimethylaminomethyl-2-propen-1-one hydrochlorides, to evaluate their antifungal activity. These designed chemical structures have an *α,β*-unsaturated ketone moiety in their chemical structures, which has crucial importance for their bioactivity. According to our knowledge, there are no studies reporting the antifungal activity of Mannich bases having acrylophenone structures. Although there are a lot of studies on the synthesis of several types of compounds by microwave irradiation, there are a very limited number of studies related to the synthesis of Mannich bases by microwave irradiation [28,29,30,31]. In this study, we also aimed to synthesize the designed compounds by two different experimental procedures, namely the conventional method and the microwave irradiation method, in order to compare the two synthetic methods used in terms of reaction time and yield of the reactions. The knowledge obtained may provide guidelines to researchers who are interested in Mannich base chemistry. In our design the aryl part was changed among phenyl, *p*-substituted phenyls and 2-thienyl. The logic to use phenyl or substituted phenyl as the aryl part of the compounds was to see how the bioactivity was affected by the electronic nature of the substituent on the phenyl ring. The logic of the replacement of the phenyl ring by a 2-thienyl in our design was to see how the bioactivity was affected by the replacement of bioisosteric rings. In this study, dimethylamine hydrochloride was used as an amine compound for the reactions. The nitrogen atom in the salt form of a Mannich base has four bonds, therefore, it has a positive inductive effect. Thus, the intermediate compound of the reaction will be stabilized by the interaction of nitrogen with oxygen (N^+^…….O^-^) or formation of hydrogen bonds between oxygen and hydrogen atoms (O…….H). These effects will increase the electrophilicity of the *β* carbon atom of Mannich bases having *α,β*-unsaturated ketone structures. It is expected that bioactivity will increase as the ratio of thiol addition increases if it is considered that the bioactivity mechanism of these compounds is thiol alkylation.

## 2. Results and Discussion

### 2.1. Chemistry

Of the compounds synthesized, compound **7** was reported for the first time. Two experimental methods were applied for the synthesis of the compounds **1-9**. They were the conventional method (method A) and the microwave irradiation method (method B). The microwave irradiation method was applied for the first time to synthesize these compounds. Physical data of the reactions is shown at Table 1 (for the conventional method), and Table 2 (for the microwave irradiation method). Antifungal activities of all compounds were also reported for the first time and these results are shown at Table 3 (disk diffusion) and Table 4 (agar dilution assay).

Some of the synthesized compounds in this study were synthesized and used as chemical intermediates for the synthesis of bis-imidazole derivatives to investigate their antifungal and antimycobacterial activities in a separate study [32]. In another study, some of these compounds were synthesized to investigate their cytotoxicity [33]. The synthetic methods used were similar in both studies. The compounds were synthesized by the conventional method in which the heating period varied from 12 h to 16 h [32]. In our study, the conventional method was one of the applied synthetic procedures and reactions were monitored by ^1^H-NMR, in addition to TLC. The reaction period was 24–72 h in the conventional method, while it was 10–120 min in the microwave irradiation method in this study. It was clear that reaction period to obtain the desired compounds was dramatically shortened by microwave irradiation. The yields of the reactions were between 30–66% by the conventional method, while they were between 15–33% by the microwave irradiation method. A common point of these two experimental methods was the production of 3-dimethylamino-1-aryl-1-propanone hydrochlorides as an additional reaction product in the reaction medium. Compounds were synthesized in higher yields in the case of conventional method, except for compound **4**. It can be suggested that microwave irradiation can be considered to make reactions in a shorter period of time. On the other hand, conventional method can be considered when the yield of the reaction is more important than reaction time. 

### 2.2. Antifungal Activity

The antifungal activity of the compounds was tested in the 3.12–200 µg/mL concentration range. Amphotericin B was used as a reference antifungal compound. The disk diffusion result and MinimalInhibition Concentration (MIC) results are shown at Table 3 and Table 4. According to the MIC results, some of the compounds had more potent antifungal activity than the reference compound amphotericin B, while others had equal antifungal activity to amphotericin B or lower activity than amphotericin B. 

The microorganism/s affected, the compounds which had more potent antifungal activity than the reference compound amphotericin B against the issued microorganism, and the ratio of potency (in paranthesis) were as follows: *Clodosporum herbarum* **6** (2), **4** (4), **2** and **5** (16); *Fusarium monifolia* **2** and **5** (8), **1**, **3**, **6**, **9** (4); *Fusarium proliferatum* **3** (2), **6** and **9** (8), **5** (16); *Fusarium solani* **2**, **6**, and **9** (4), **5** (8); *Peacilomyces sulpherolloides* **6** and **9** (2), **2** and **5** (4); *Penicillum expansum* (lemon) **2**, **5** and **6** (2); *Penicillum expansum* (Clinic) **2**, **5**, **6**, and **9** (4); *Penicillum italicum*’a **2**, **3**, **4**, **5**, and **9** (2). 

The compounds which had equal antifungal activity with the reference compound amphotericin B, and the microorganism affected were as follows: **2** against *Alternaria alternata*; **1**, **3**, and **9** against *Clodosporum herbarum*; **1** and **4** against *Fusarium proliferatum* and *Fusarium solani*; **1** and **3** against *Peacilomyces sulpherolloides*; **3** and **9** against *Penicillum expansum* (lemon); **3** against *Penicillum expansum* (Clinic).

The microorganism/s affected and the most effective compound/s against them were as follows: *Alternaria alternata* (**2**), *Clodosporum herbarum, Fusarium monifolia* and *Peacilomyces sulpherolloides* (**2**, **5**), *Fusarium proliferatum* and *Fusarium solani* (**5**), *Penicillum expansum* (lemon) (**2**, **5**, **6**), *Penicillum expansum* (Clinic) (**2**, **5**, **6**, **9**), *Penicillum italicum* (**2**, **3**, **4**, **5**, **9**). None of the compounds showed antifungal activity against *Rhizopus* sp. and *Trichoderma harzianum* in the concentration range studied. Although some of the compounds were effective against *Aspergillus fumigatus*, *Aspergillus niger*, *Phoma* sp. and Mucor sp., their effectiveness were lower than the reference compound amphotericin B. Bioactivity was not dependent on electronic nature of the substituent on phenyl ring.

The reported mechanism of the antifungal and/or cytotoxic activities of Mannich bases in the literature is thiol alkylation [2,7,23,24,25,34,35,36,37]. There is a reported stability study, which was realized with the compound **8** in our study [37]. That study supports that the antifungal activity mechanism of the synthesized compounds may indeed be thiol alkylation, since the compound **8** produced the corresponding thiol adduct. The compounds synthesized in this study most probably display their antifungal activity by alkylation of the thiol groups of proteins in fungi.

**Table 1:** Physical data of the reactions by conventional method.

Compound	Ar	Ketone (mmol)	Paraformaldehyde (mmol)	Dimethylamine hydrochloride (mmol)	Acetic acid (mL)	Time (hours)	Yield %
**1**	C_6_H_5_	17	34	17	43	24	62
**2**	4-CH_3_C_6_H_4_	15	30	15	38	24	33
**3**	4-CH_3_OC_6_H_4_	13	26	13	33	48	62
**4**	4-ClC_6_H_4_	13	26	13	33	24	25
**5**	4-FC_6_H_4_	14	28	14	35	24	66
**6**	4-BrC_6_H_4_	10	20	10	25	24	30
**7**	4-HOC_6_H_4_	15	30	15	38	72	53
**8**	4-NO_2_C_6_H_4_	12	24	12	30	24	45
**9**	C_4_H_3_S(2-yl)	16	32	16	40	24	48

**Table 2:** Physical data of the reactions by microwave irradiation method.

Compound	Ar	Ketone (mmol)	Paraformaldehyde (mmol)	Dimethylamine hydrochloride (mmol)		Microwave Condition P (t)^a^		Crystallization solvent	Yield (%)
**1**	C_6_H_5_	4.2	8.4		4.2		70W (60 min)	EtOAc/ Et_2_O	24
**2**	4-CH_3_C_6_H_4_	3.7	7.4		3.7		70W(120 min)	MeOH/ Et_2_O	28
**3**	4-CH_3_OC_6_H_4_	3.3	6.6		3.3		70W(100 min)	Acetone/ Et_2_O	26
**4**	4-ClC_6_H_4_	3.2	6.4		3.2		70W(10 min)	EtOH/ Et_2_O	33
**5**	4-FC_6_H_4_	3.6	7.2		3.6		70W(40 min)	EtOH/ Et_2_O	32
**6**	4-BrC_6_H_4_	2.5	5.0		2.5		70W(70 min)	EtOAc/ Et_2_O	21
**7**	4-HOC_6_H_4_	3.7	7.4		3.7		70W(120 min)	MeOH/ Et_2_O	15
**8**	4-NO_2_C_6_H_4_	6.0	12.0		6.0		70W(50 min)	MeOH/ Et_2_O	37
**9**	C_4_H_3_S(2-yl)	7.9	15.8		7.9		70W(10 min)	MeOH/ Et_2_O	30

^a^ P: Power used (Watt, W), t: Irradiation time (Minutes, min).

**Table 3:** Antifungal activity of synthesized compounds against fungi isolates. The test was based on disk diffusion method.

Compound	1	2	3	4	5	6	7	8	9
Fungi	DD^a^	DD^a^	DD^a^	DD^a^	DD^a^	DD^a^	DD^a^	DD^a^	DD^a^
*Alternaria alternata* TK1	11	26	18	9	21	14			16
*Aspergillus fumigatus* TK2		14	13		12	11			9
*Aspergillus niger* TK3	8	8	10			10			10
*Clodosporum herbarum* TK6	7	19	7	9	13	10			8
*Fusarium monifolia* TK7	13	21	14	10	18	20			15
*Fusarium proliferatum* TK8	11	21	13	9	18	14			17
*Fusarium solani* TK9	13	19	10	12	20	16			17
*Mucor* sp. TK11	13	18	17		16				17
*Peacilomyces sulpherolloides* TK21	20	34	18	12	26	26	10	10	23
*Penicillum expansum* (lemon) TK13		10	7		9	10			8
*Penicillum expansum* (Clinic) TK14		21	14		15	19			12
*Penicillum italicum* TK15	14	32	28	26	40	50	12	10	38
*Phoma* sp. TK20		9	9		9	10			9
*Rhizopus* sp. TK17									
*Trichoderma harzianum* TK18									

^a^ DD = Inhibition diameter for sample 300 µg/disc (mm).

**Table 4:** Minimal Inhibition Concentration **(**MIC) values of synthesized compounds against fungi isolates tested in agar dilution assay (*µ*g/mL).

Compound	1	2	3	4	5	6	7	8	9	Amp B^a^
Fungi										
*Alternaria alternata* TK1	12.5	3.12	6.25	50	6.25	6.25			12.5	3.12
*Aspergillus fumigatus* TK2		12.5			12.5	50			100	6.25
*Aspergillus niger* TK3	200	200	100			100			100	12.5
*Clodosporum herbarum* TK6	200	12.5	200	50	12.5	100			200	200
*Fusarium monifolia* TK7	12.5	6.25	12.5	100	6.25	12.5			12.5	50
*Fusarium proliferatum* TK8	100		50	100	6.25	12.5			12.5	100
*Fusarium solani* TK9	50	12.5	100	50	6.25	12.5			12.5	50
*Mucor* sp. TK11	25	12.5	25		12.5				12.5	6.25
*Peacilomyces sulpherolloides* TK21	12.5	3.12	12.5	50	3.12	6.25	100	200	6.25	12.5
*Penicillum expansum* (lemon) TK13		100	200		100	100			200	200
*Penicillum expansum* (Clinic) TK14		12.5	50		12.5	12.5			12.5	50
*Penicillum italicum* TK15	12.5	3.12	3.12	3.12	3.12	50	50	100	3.12	6,25
*Phoma* sp. TK20		100	100		200	200			200	50
*Rhizopus* sp. TK17										3,12
*Trichoderma harzianum* TK18										3,12

^a^ Amp B: amphotericin B.

To understand how the replacement of the phenyl ring by its bioisoster thiophene ring affects the antifungal activity, the antifungal activities of the compounds **1** and **9** against fungi, except *Rhizopus* sp. and *Trichoderma harzianum*, were compared. Both compounds had equal antifungal activity against *Alternaria alternata*, *Clodosporum herbarum* and *Fusarium monifolia*. It means that the replacement of the rings did not affect the activity in this case. On the other hand, the replacement of the phenyl ring by thiophene ring increased the antifungal activity two-fold against *Aspergillus niger*, *Mucor* sp., and *Peacilomyces sulpherolloides*; four-fold against *Fusarium solani* and *Penicillum italicum*, and eight-fold against *Fusarium proliferatum*. However, compound **1**, which has a phenyl ring, was ineffective against *Aspergillus fumigatus*, *Penicillum expansum* (lemon), *Penicillum expansum* (Clinic), and *Phoma* sp. in the concentration range studied, while compound **9**, which has thiophene ring, had shown activity at 12.5–200 µg/mL against the same fungi. According to the results obtained, it can be said that the effect of the replacement of the phenyl ring by its bioisoster thiophene ring on antifungal activity of the compounds was not consistent. 

To conclude, the compounds **1-6**, and **9**, which had more potent (2–16 times) antifungal activity than the reference compound amphotericin B against some fungi, can be model compounds for further studies to develop new antifungal agents. In addition, microwave irradiation can be considered to reduce reaction time, while conventional method can be considered to obtain compounds with higher reaction yields. 

## 3. Experimental

### 3.1. Chemistry

Chemicals used in this study were as follows: acetophenone, 4'-methylacetophenone, 4'-nitroaceto-phenone, 4'-chloroacetophenone, 2-acetylthiophene (Fluka, Steinheim, Switzerland), 4'-methoxy-acetophenone, 4'-fluoroacetophenone, 4'-bromoacetophenone (Acros, Geel, Belgium), 4'-hydroxy-acetophenone (Merck, Hohenbrunn, Germany), paraformaldehyde (Merck, Darmstadt, Germany), methanol, ethyl acetate (Riedel-deHaën, Seelze, Germany), diethyl ether (Fluka, Steinheim, Switzerland), ethanol, acetone (J. T. Baker, Deventer, Holland), acetic acid (Merck, Darmstadt, Germany), and amphotericin B (Sigma**–**Aldrich, Taufkirchen, Germany). Melting points were determined on a Büchi 530 (Flawil, Switzerland). The ^1^H- and ^13^C-NMR spectra were recorded at 200 (50) MHz on a Gemini-Varian spectrometer (Danbury, CT, USA). High resolution mass spectrum (HRMS) of compound **7** was recorded on a HPLC-TOF Waters Micromass LCT Premier XE (Milford, MA, USA) mass spectrometer using an electrospray ion source (ESI).

### 3.2. Synthesis of 1-Aryl-2-dimethylaminomethyl-2-propen-1-one Hydrochlorides (Figure 1)

Compounds were synthesized by two experimental ways, namely the conventional method (method A) and the microwave irradiation method (method B). Reactions were followed by TLC and ^1^H-NMR. Chemical structures of the compounds synthesized were confirmed by reported ^1^H-NMR and melting points [32]. They were in accordance with the reported literature values. For compound **7**, which was reported for the first time, ^13^C-NMR and HRMS were also taken additionally.

**Figure 1:**
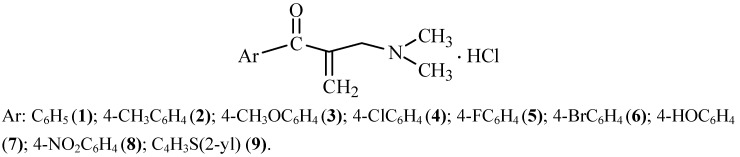
Chemical structures of the synthesized compounds **1**-**9**.

#### 3.2.1. Conventional Method (Method A)

To a solution of suitable ketone in acetic acid, paraformaldehyde and dimethylamine hydrochloride were added. The mole ratios of the reagents used were in 1:2:1, respectively. The mixture was heated under stirring for several hours. Thereafter, the solvent was removed under reduced pressure. The crude compounds obtained were crystallized from methanol-diethyl ether. Physical data of the reactions by conventional method such as the amounts (mmol) of ketone, paraformaldehyde and dimethylamine hydrochloride used, acetic acid (mL), reflux times (hours), and yields of the reactions (%) were summarized at Table 1. ^1^H-NMR, ^13^C-NMR and HRMS results of the compound **7**, which is a new compound, were as follows: ^1^H-NMR (200 MHz, DMSO-d_6_) δ 2.76 (s, 6H, (CH_3_)_2_N), 4.07 (s, 2H, CH_2_), 6.11 (s, 1H, =CH_2_), 6.57 (s, 1H, =CH_2_), 6.94 (d, 2H, arom., *J* = 8.8 Hz), 7.75 (d, 2H, arom., *J* = 8.8 Hz), 10.51 (br s, 1H, OH), 10.72 (br s, 1H, NH^+^). ^13^C-NMR (50 MHz, DMSO-d_6_) δ 44.0, 58.2, 117.2, 128.4, 134.2, 136.1, 138.7, 164.4, 195.5. HRMS (ESI^+^) calcd for C_12_H_15_NO_2_ [M+H^+^] 206.1181, found 206.1171. Melting point of compound **7** was 186–8 °C. 

#### 3.2.2. Microwave Irradiation Method (Method B)

Suitable ketone, paraformaldehyde and dimethylamine hydrochloride in 1:2:1, respectively, were heated in acetic acid at 120 °C and at 70 Watt for 10–120 minutes. Reactions were monitored by TLC and ^1^H-NMR. Irradiation power and reaction time were changed accordingly to obtain the compounds with possible maximal yield this situation was shown at Table 2. When the reaction was stopped, solvent was removed and crude compound was purified by crystallization from suitable solvent. The physical data of the reactions by microwave irradiation method such as the amounts (mmol) of ketone, paraformaldehyde and dimethylamine hydrochloride used, microwave condition (power used (watt) and irradiation time (minute)), crystallization solvents and yields of the reactions (%) were summarized in Table 2.

### 3.3. Antifungal Activity Assay

#### 3.3.1. Microbial strains

The antifungal activities of the synthesized compounds against 15 fungi species were tested by using disc-diffusion and Minimal Inhibition Concentration **(**MIC) Agar Dilution Assay [5,38,39]. The list of microorganisms used in this study is given in Table 3 and Table 4. Microorganisms were provided by the Department of Biology, Ataturk University, Erzurum, Turkey. Identity of the microorganisms used in this study was confirmed by Microbial Identification System in Biotechnology Application and Research Center at Ataturk University.

#### 3.3.2. Disc Diffusion Assay

The samples were dissolved in 10% dimethyl sulfoxide (DMSO) to a final concentration of 30 mg/mL and sterilized by filtration with 0.22 µm Millipore filters. Antifungal tests were then carried out by a disc diffusion method using 100 µL of suspension containing 10^4^ spore/mL of fungi spread on potato dextrose agar (PDA) medium. The discs (6 mm in diameter) were impregnated with 10 µL of the samples (300 µg/disc) at a concentration of 30 mg/mL and placed on the inoculated agar. Negative controls were prepared using 10% DMSO. The inoculated plates were incubated at 37 °C 72 h for fungi isolates. Plant associated microorganisms were incubated at 27 °C. Antifungal activity was evaluated by measuring the zone of inhibition against the test organisms. Each assay in this experiment was repeated twice [38]. 

#### 3.3.3. MIC Agar Dilution Assay

Minimal Inhibition Concentration **(**MIC) values of the fungi isolates were studied based on the agar dilution method. The samples were added aseptically to sterile molten PDA medium, containing Tween 20 (Sigma 0.5%, v/v), at the appropriate volume to produce the concentration range of 3.12–200 µg/mL. The resulting PDA solutions were immediately poured into Petri plates after vortexing. The plates were spot inoculated with 5 µL (10^4^ spore/mL) of each fungal isolate. Amphotericin B (Sigma A 4888) was used as a reference antifungal drug since it is a clinally used drug as used in our earlier studies [2,3,4,5]. The inoculated plates were incubated at 27 and 37 °C for 72 h for plant and clinical fungi isolates, respectively. At the end of the incubation period, the plates were evaluated for the presence or absence of growth. MIC values were determined as the lowest concentration of the synthesized compounds where the absence of growth was recorded. Each test was repeated at least twice [39]. 

## 4. Conclusions

Compounds **1-6**, and **9**, which had more potent (2–16 times) antifungal activity than the reference compound amphotericin B against some fungi, can be model compounds for further studies to develop new antifungal agents. In addition, microwave irradiation can be considered to reduce reaction period, while the conventional method can still be considered to obtain compounds with higher reaction yields in the synthesis of new Mannich bases.
